# Supplementary material for: Postsynaptic plasticity of Purkinje cells in mice is determined by molecular identity
Source: Commun Biol. 2022 Dec 3;5:1328. doi: 10.1038/s42003-022-04283-y (PMC9719509; doi:10.1038/s42003-022-04283-y)
Supplement: Supplementary file 2 — Supplementary Information [file 42003_2022_4283_MOESM2_ESM.pdf]

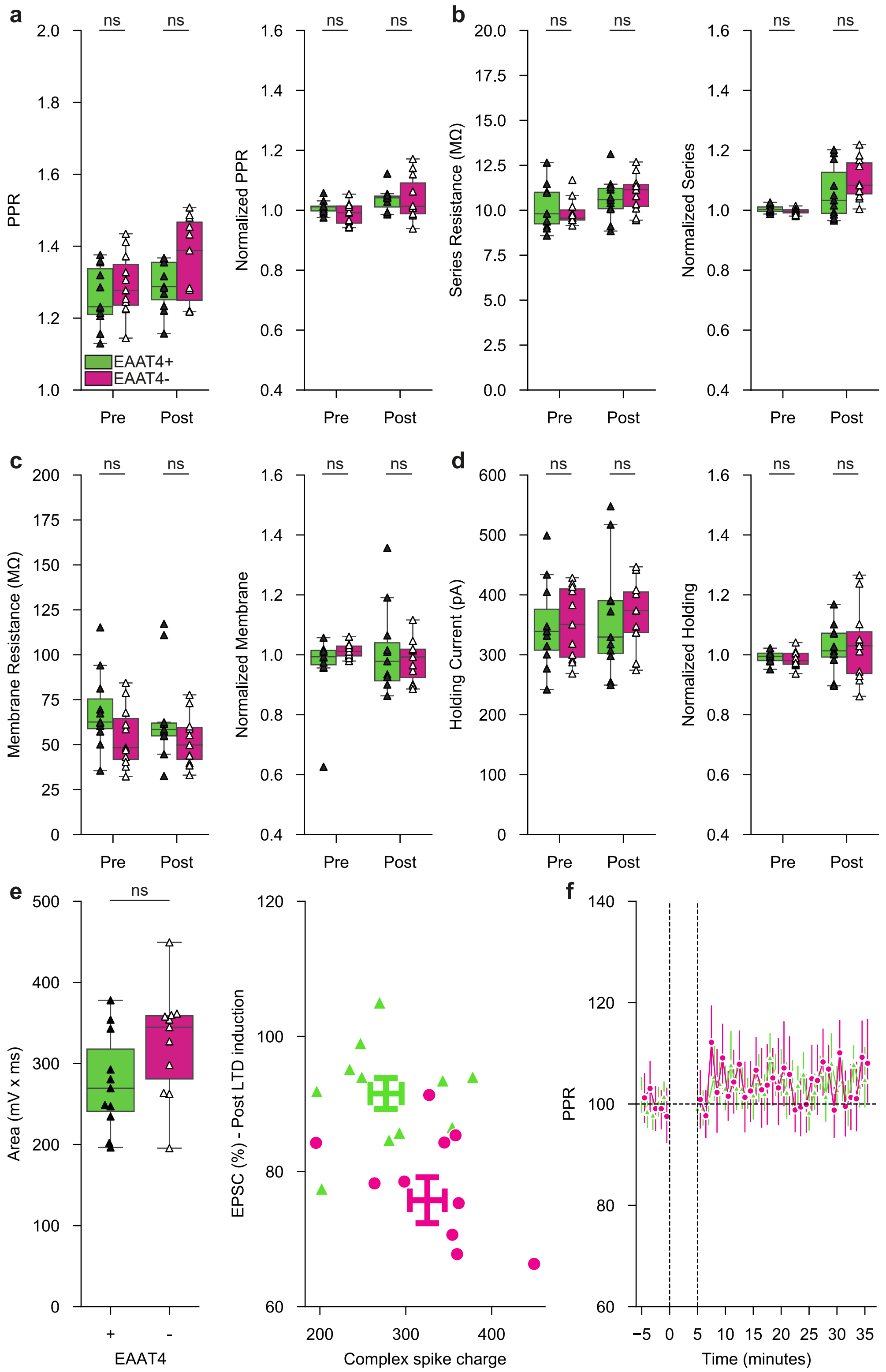

Supplementary Fig. 1 | No differences are present in additional recorded properties of PCs subjected to postsynaptic LTD induction. **a,b,c,d,** Means of pre and post LTD induction. **a,** The paired pulse ratio (PPR) pre and post LTD induction (left) and normalized PPR (right). **b,** Pipette series resistance pre and post LTD induction (left) and normalized (right). **c,** Purkinje cell membrane resistance pre and post LTD induction (left) and normalized membrane resistance (right). **d,** Holding current required to keep membrane voltage at -65 mV in voltage clamp pre and post LTD induction (left) and normalized holding current (right). **e,** Average integrated area of the complex spike and spikelets during LTD induction (left). Scatterplot of the change in EPSC size post LTD induction EAAT4+ (green) and EAAT4- (magenta) PCs, and the size of the CS (right). We find no significant correlation between change in EPSC size and size of the CS for both EAAT4+ (p = 0.82) and EAAT4- (p = 0.73) groups. Correlations were asessed with a Pearson-r test. Crosses represent the mean value of the populations. Error bars represent the standard error. **f,** Normalized time-course of the PPR (EPSC1/EPSC2) before (-5 to 0) and after (5 to 35) LTD induction in EAAT4+ and EAAT4- PCs. Independent samples t-test as used to determine differences for all statistical data presented in this figure (Suppl. Table 1). For all panels EAAT4+ n = 11, EAAT4- n = 11.

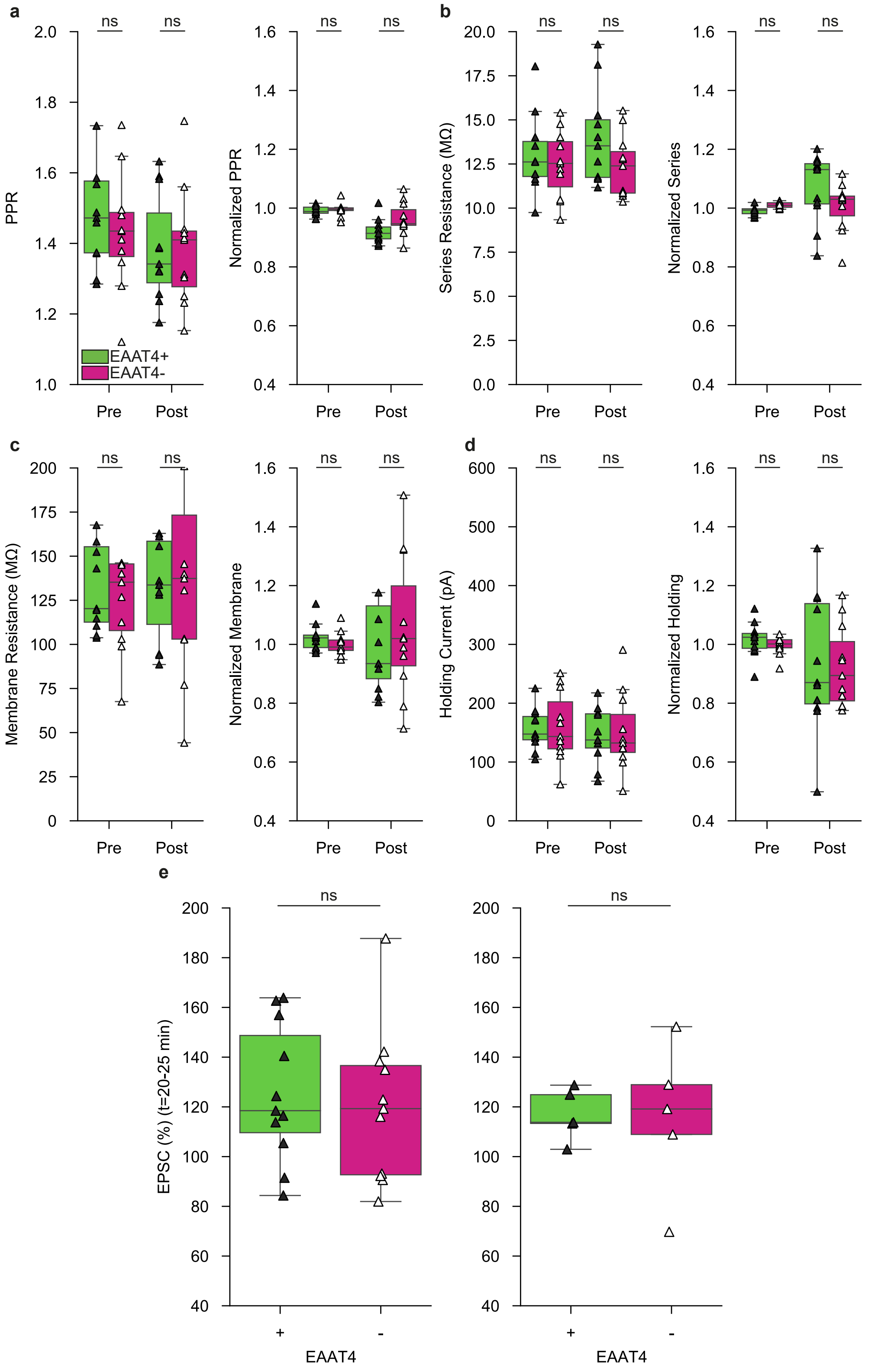

Supplementary Fig. 2 | No differences are present in additional recorded properties of PCs subjected to presynaptic LTP induction. a,b,c,d, Means of pre and post LTP induction. a, The paired pulse ratio (PPR) pre and post LTP induction (left) and normalized PPR (right). **b,** Pipette series resistance pre and post LTP induction (left) and normalized (right). **c,** Purkinje cell membrane resistance pre and post LTP induction (left) and normalized membrane resistance (right). **d,** Holding current required to keep membrane voltage at -65 mV in voltage clamp pre and post LTP induction (left) and normalized holding current (right). **e,** Mean normalized EPSC size 20-25 minutes post LTP induction in sagittal slices at room temperature (left) and in coronal slices at physiological temperature. Independent samples t-test as used to determine differences for all statistical data presented in this figure (Suppl. Table 2). For all panels except e (right) EAAT4+ n = 11, EAAT4- n = 11. For panel e (right) EAAT4+ n = 5, EAAT4- n = 5.

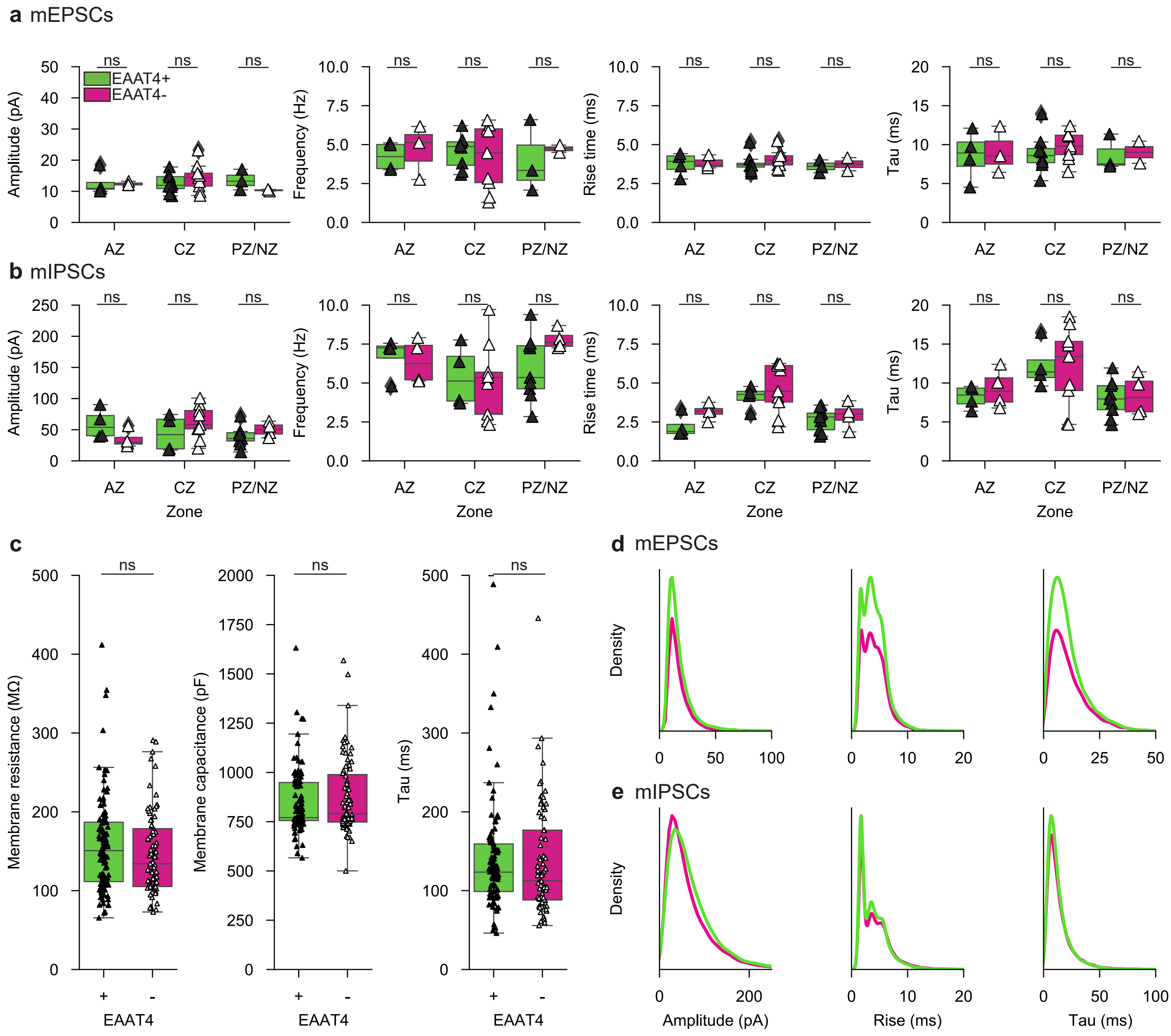

Supplementary Fig. 3 | Additional data on mEPSCs and mIPSCs. a, From left to right: Amplitude, frequency, rise time, and decay time (tau) of mEPSCs of EAAT4+ (green) and EAAT4- (red) PCs divided by cerebellar region (anterior zone (AZ, n = 4 EAAT4+, 2 EAAT4-), central zone (CZ, n = 8 EAAT4+, 9 EAAT4-), and posterior/nodular zone (PZ/NZ, n = 3 EAAT4+, 2 EAAT4-)). **b,** Same as for a, but for mIPSCs. (AZ, n = 4 EAAT4+, 4 EAAT4-) (CZ n = 4 EAAT4+, 8 EAAT4-) (PZ/NZ, n = 9 EAAT4+, 4 EAAT4-) **c,** Additional membrane properties of cells recorded from both the mEPSC and mIPSCs dataset combined. We observe no difference in membrane resistance, capacitance, or Tau (Membrane resistance x capacitance). EAAT4+ n = 101, EAAT4- n = 75. **d, e** Distribution of the amplitude, rise time, and decay time of mEPSCs **(d)** and mIPSCs **(e)**. Error bars represent the 25 percentile. Differences between the amplitudes of mEPSCs and mIPSCs in different zones were determined with a Mann-Whitney U-test. All other data were assessed for differences with independent samples t-tests.

Supplementary Table 1: Table containing additional statistics of the performed LTD experiments. P-values from Mann-Whitney U-tests (MW-U) and independent samples t-test (Ind-ttest) results are shown, where applicable. Values are shown as n, mean, and standard error (SEM), respectively.

LTD	EAAT4+			EAAT4-			P-value	
	n	Mean	SEM	n	Mean	SEM	Ind-ttest	MW-U
LTD	11	0.92	0.02	11	0.75	0.02	0.00085	-
LTD – Paired lobules	5	0.94	0.02	10	0.77	0.04	-	0.002
LTD - 25 minutes	11	0.91	0.03	11	0.77	0.04	0.004	-
LTD - PPR (pre-induction)	11	1.25	0.02	11	1.30	0.03	0.185	-
LTD - PPR (post-induction)	11	1.28	0.02	11	1.33	0.03	0.147	-
LTD - Rs (pre-induction)	11	10.06	0.39	11	9.94	0.23	0.802	-
LTD - Rs (post-induction)	11	10.51	0.37	11	10.85	0.29	0.474	-
LTD - Rm (pre-induction)	11	62.5	4.9	11	53.5	4.8	0.201	-
LTD – Rm (post-induction)	11	64.5	7.2	11	52.1	4.3	0.156	-
LTD - Holding (pre-induction)	11	350.4	22.8	11	357.4	19.3	0.818	-
LTD - Holding (post-induction)	11	362.2	29.4	11	360.7	17.2	0.966	-
LTD - Complex spike	11	277.0	18.2	11	325.0	20.3	0.094	-

Supplementary Table 2: Table containing statistics of the performed LTP experiments. P-values from Mann-Whitney U-tests (MW-U) and independent samples t-test (Ind-ttest) results are shown. Values are shown as n, mean, and standard error (SEM), respectively.

LTP	EAAT4+			EAAT4-			P-value	
	n	Mean	SEM	n	Mean	SEM	Ind-ttest	MW-U
LTP	11	1.16	0.08	11	1.13	0.09	0.803	-
LTP - 25 minutes	11	1.19	0.10	11	1.24	0.11	0.731	-
LTP - PT	5	1.17	0.05	5	1.15	0.15	0.902	-
LTP - PPR (pre-induction)	11	1.50	0.05	11	1.43	0.05	0.372	-
LTP - PPR (post-induction)	11	1.38	0.04	11	1.37	0.05	0.913	-
LTP - Rs (pre-induction)	11	13.17	0.72	11	12.34	0.56	0.373	-
LTP - Rs (post-induction)	11	13.90	0.77	11	12.28	0.53	0.097	-
LTP - Rm (pre-induction)	11	150.8	23.2	11	136.7	14.1	0.61	-
LTP - Rm (post-induction)	11	163.9	40.6	11	153.5	25.9	0.832	-
LTP - Holding (pre-induction)	11	156.4	12.2	11	159.7	18.8	0.886	-
LTP - Holding (post-induction)	11	143.7	14.4	11	148.3	19.9	0.854	-

Supplementary Table 3: Table containing statistics of the performed miniature postsynaptic current experiments. P-values from Mann-Whitney U-tests (MW-U) and independent samples t-test (Ind-ttest) results are shown. Values are shown as n, mean, and standard error (SEM), respectively.

Miniatures	EAAT4+			EAAT4-			P-value	
	n	Mean	SEM	n	Mean	SEM	Ind-ttest	MW-U
mEPSC - Amplitude	40	14.7	0.89	26	14.3	0.80	-	0.33
mEPSC - Frequency	40	4.29	0.25	26	4.31	0.32	0.956	-
mEPSC - Rise time	40	3.84	0.09	26	3.85	0.10	0.939	-
mEPSC - Tau	40	9.00	0.37	26	9.04	0.40	0.926	-
mIPSC - Amplitude	46	43.8	3.4	42	44.6	3.8	-	0.498
mIPSC - Frequency	46	5.39	0.31	42	5.41	0.31	0.962	-
mIPSC - Rise time	46	3.40	0.14	42	3.83	0.19	0.064	-
mIPSC - Tau	46	10.29	0.45	42	11.27	0.57	0.177	-
