# Supplementary material for: Postsynaptic plasticity of Purkinje cells in mice is determined by molecular identity
Source: Commun Biol. 2022 Dec 3;5:1328. doi: 10.1038/s42003-022-04283-y (PMC9719509; doi:10.1038/s42003-022-04283-y)
Supplement: Supplementary file 3 — Description of Additional Supplementary Files [file 42003_2022_4283_MOESM3_ESM.docx]

**Description of Additional Supplementary Files**

**File name:** Supplementary Data 1
**Description:** Raw data trace describing the expression of EAAT4 as shown in figure 1b.

**File name:** Supplementary Data 2
**Description:** Raw data trace describing the expression of PLCB4 as shown in figure 1b.

**File name:** Supplementary Data 3
**Description:** Raw data trace describing the expression of Aldolase C as shown in figure 1b.

**File name:** Supplementary Data 4
**Description:** Dataset from the LTD experiments containing data from each cell averaged per minute, pre and post LTD induction.

**File name:** Supplementary Data 5
**Description:** Dataset from the LTP experiments at room temperature containing data from each cell averaged per minute, pre and post LTP induction.

**File name:** Supplementary Data 6
**Description:** Dataset from the LTP experiments at physiological temperature containing data from each cell averaged per minute, pre and post LTP induction.

**File name:** Supplementary Data 7
**Description:** Average values from each cell of the mEPSC experiments.

**File name:** Supplementary Data 8
**Description:** Dataset containing values from each individual mEPSC event.

**File name:** Supplementary Data 9
**Description:** Average values from each cell of the mIPSC experiments.

**File name:** Supplementary Data 10
**Description:** Dataset containing values from each individual mIPSC event.

**File name:** Supplementary Data 11
**Description:** Dataset with 'seal' values from the miniature experiments. Contains data from both mEPSC and mIPSC datasets.
