# Supplementary material for: Postsynaptic plasticity of Purkinje cells in mice is determined by molecular identity
Source: Commun Biol. 2022 Dec 3;5:1328. doi: 10.1038/s42003-022-04283-y (PMC9719509; doi:10.1038/s42003-022-04283-y)
Supplement: Supplementary file 15 — Reporting Summary [file 42003_2022_4283_MOESM15_ESM.pdf]

nature portfolio

Corresponding author(s): S. Voerman

Last updated by author(s): Nov 8, 2022

Reporting Summary

Nature Portfolio wishes to improve the reproducibility of the work that we publish. This form provides structure for consistency and transparency in reporting. For further information on Nature Portfolio policies, see our Editorial Policies and the Editorial Policy Checklist.

Statistics

For all statistical analyses, confirm that the following items are present in the figure legend, table legend, main text, or Methods section.

n/a	Confirmed	
☐	☒	The exact sample size (*n*) for each experimental group/condition, given as a discrete number and unit of measurement
☐	☒	A statement on whether measurements were taken from distinct samples or whether the same sample was measured repeatedly
☐	☒	The statistical test(s) used AND whether they are one- or two-sided<br>*Only common tests should be described solely by name; describe more complex techniques in the Methods section.*
☐	☒	A description of all covariates tested
☐	☒	A description of any assumptions or corrections, such as tests of normality and adjustment for multiple comparisons
☐	☒	A full description of the statistical parameters including central tendency (e.g. means) or other basic estimates (e.g. regression coefficient) AND variation (e.g. standard deviation) or associated estimates of uncertainty (e.g. confidence intervals)
☐	☒	For null hypothesis testing, the test statistic (e.g. F, t, r) with confidence intervals, effect sizes, degrees of freedom and P value noted<br>*Give P values as exact values whenever suitable.*
☒	☐	For Bayesian analysis, information on the choice of priors and Markov chain Monte Carlo settings
☒	☐	For hierarchical and complex designs, identification of the appropriate level for tests and full reporting of outcomes
☒	☐	Estimates of effect sizes (e.g. Cohen's d, Pearson's r), indicating how they were calculated

Our web collection on statistics for biologists contains articles on many of the points above.

Software and code

Policy information about availability of computer code

Data collection	No custom or open source software was used for data collection.
Data analysis	All data were analyzed using custom Python scripts (Python 3.7+).<br>Electrophysiological data was imported into Python using Neural Ensemble (https://neuralensemble.org/neo/)<br>mEPSCs and mIPSCs were automatically analyzed using a deconvolution-based method (https://www.ncbi.nlm.nih.gov/pmc/articles/PMC3471482/)<br>All code is available in a github repository (https://github.com/s-voerman/EAAT4-Project)

For manuscripts utilizing custom algorithms or software that are central to the research but not yet described in published literature, software must be made available to editors and reviewers. We strongly encourage code deposition in a community repository (e.g. GitHub). See the Nature Portfolio guidelines for submitting code & software for further information.

Data

Policy information about availability of data

All manuscripts must include a data availability statement. This statement should provide the following information, where applicable:

- Accession codes, unique identifiers, or web links for publicly available datasets
- A description of any restrictions on data availability
- For clinical datasets or third party data, please ensure that the statement adheres to our policy

Data and codes will be made available upon request to the corresponding author.

Human research participants

Policy information about studies involving human research participants and Sex and Gender in Research.

Reporting on sex and gender	N/A
Population characteristics	N/A
Recruitment	N/A
Ethics oversight	N/A

Note that full information on the approval of the study protocol must also be provided in the manuscript.

Field-specific reporting

Please select the one below that is the best fit for your research. If you are not sure, read the appropriate sections before making your selection.

☒ Life sciences ☐ Behavioural & social sciences ☐ Ecological, evolutionary & environmental sciences

For a reference copy of the document with all sections, see nature.com/documents/nr-reporting-summary-flat.pdf

Life sciences study design

All studies must disclose on these points even when the disclosure is negative.

Sample size	No prior calculation of sample sizes were performed. Sample sizes were based on the number of samples used in similar publications.
Data exclusions	Data in plasticity datasets (LTD/LTD) were excluded if Ri changed by >25% over the course of the experiments, or if EPSC size was already trending positively or negatively before induction of long term plasticity.<br>Data was excluded from mEPSC and mIPSC datasets if the ratio between series and input resistance was > 15% or if holding current was > -500 pA. Additionally, PCs were excluded if the frequency of miniatures was extremely low (< 0.5 Hz) 223 or extremely high (>15 Hz).
Replication	No measures were taken to verify the reproducibility of the findings.
Randomization	Samples (individual PCs) were assigned into either EAAT4+ or EAAT4- groups based on their expression when recording in vitro. These samples were not randomized.
Blinding	No blinding was performed, as it was necessary for the researchers to be able to target Purkinje cells with specific levels of EAAT4 expression.

Reporting for specific materials, systems and methods

We require information from authors about some types of materials, experimental systems and methods used in many studies. Here, indicate whether each material, system or method listed is relevant to your study. If you are not sure if a list item applies to your research, read the appropriate section before selecting a response.

Materials & experimental systems

n/a	Involved in the study
☐	☒ Antibodies
☒	☐ Eukaryotic cell lines
☒	☐ Palaeontology and archaeology
☐	☒ Animals and other organisms
☒	☐ Clinical data
☒	☐ Dual use research of concern

Methods

n/a	Involved in the study
☒	☐ ChIP-seq
☒	☐ Flow cytometry
☒	☐ MRI-based neuroimaging

Antibodies

Antibodies used

Primary Antibodies used in IHC:
Anti-PLCB4 | Rabbit | SC-20760 | Santa Cruz Biotechnology | (1:500)
Anti-AldolaseC | Guinea Pig | 464004 | Synaptic Systems | (1:1000)
anti-GFP | Chicken | GFP-1020 |Aveslabs | (1:500)

Secondary Antibodies used in IHC:
Streptavidin Cy3 | AB_2337244 | Jackson ImmunoResearch | (1:400)
Rabbit Cy3 | AB_2307443 | Jackson ImmunoResearch | (1:400)
Guinea Pig AF-647 | AB_2340476 | Jackson ImmunoResearch | (1:400)
Chicken AF-488 | AB_2340375| Jackson ImmunoResearch | (1:400)

Biocytin was sourced from Sigma-Aldrich (B4261) and added to internal solution at a concentration of 1 mg/ml

Validation

The primary antibodies used in this study were validated either by manufacturers or by previously performed studies.

Animals and other research organisms

Policy information about studies involving animals; ARRIVE guidelines recommended for reporting animal research, and Sex and Gender in Research

Laboratory animals

Mice, EAAT4-eGFP-reporter, C57Bl6/Jax background, aged >p40
Mice, C57Bl6/Jax, aged >p40

Wild animals

N/A

Reporting on sex

Findings are not related to the sex of the animal. Both male and female animals were used in this study, and sex was not considered in study design.

Field-collected samples

N/A

Ethics oversight

All animal experiments were performed in accordance with the guidelines of the Dutch Ethical Committee for animal experiments, and in accordance with the Animal Welfare Board of the Erasmus MC, in line with Dutch and EU legislation.

Note that full information on the approval of the study protocol must also be provided in the manuscript.
